# A Study of Dispersion Compensation of Polarization Multiplexing-Based OFDM-OCDMA for Radio-over-Fiber Transmissions

**DOI:** 10.3390/s16091440

**Published:** 2016-09-07

**Authors:** Chih-Ta Yen, Wen-Bin Chen

**Affiliations:** Department of Electrical Engineering, National Formosa University, Yunlin County 632, Taiwan; energy00417@hotmail.com

**Keywords:** spectral-amplitude-coding optical code-division multiple-access (SAC-OCDMA), orthogonal frequency-division multiplexing (OFDM), balanced photo-detector (BPD), multiple access interference (MAI), dispersion equalize

## Abstract

Chromatic dispersion from optical fiber is the most important problem that produces temporal skews and destroys the rectangular structure of code patterns in the spectra-amplitude-coding-based optical code-division multiple-access (SAC-OCDMA) system. Thus, the balance detection scheme does not work perfectly to cancel multiple access interference (MAI) and the system performance will be degraded. Orthogonal frequency-division multiplexing (OFDM) is the fastest developing technology in the academic and industrial fields of wireless transmission. In this study, the radio-over-fiber system is realized by integrating OFDM and OCDMA via polarization multiplexing scheme. The electronic dispersion compensation (EDC) equalizer element of OFDM integrated with the dispersion compensation fiber (DCF) is used in the proposed radio-over-fiber (RoF) system, which can efficiently suppress the chromatic dispersion influence in long-haul transmitted distance. A set of length differences for 10 km-long single-mode fiber (SMF) and 4 km-long DCF is to verify the compensation scheme by relative equalizer algorithms and constellation diagrams. In the simulation result, the proposed dispersion mechanism successfully compensates the dispersion from SMF and the system performance with dispersion equalizer is highly improved.

## 1. Introduction

Optical code-division multiple-access (OCDMA) schemes allow multiple users in a local area network (LANs) to access the same fiber channel asynchronously without delays or the need for scheduling. Accordingly, OCDMA represents an attractive access technique for radio-over-fiber (RoF) networks [1,2]. In the RoF system, micro-cells are connected by optical fibers to radio base stations (RBSs), which are connected in turn to central control stations (CSs). Regarding the configuration of RoF networks, a linear bus or passive star optical link is generally preferable since architecture of this type lowers the cost of implementation and enables the straightforward addition of additional access points. In a RoF network with OCDMA access, different RBSs assign different codes. The advantage of the network is easily to add/remove the access points by changing the length of codes.

In the past decade, spectral-amplitude-coding OCDMA (SAC-OCDMA) systems have gained increasing attention. When SAC-OCDMA systems are coupled with quasi-orthogonal code, multiple access interferences (MAI) can be eradicated in ideal conditions, rendering SAC-OCDMA systems extremely suitable for RoF systems. The SAC-OCDMA is the most appropriate way for signal transmitting because it is uncomplicated and the information data can be regenerated without any synchronization and can be implemented in analog intensity modulation (IM) radio links. The detailed operational theorem of the decoding scheme has been proposed in [3,4].

The other OCDMA scheme is coding in polarized domain. In spectral polarization coding architectures, light sources are divided into two mutually orthogonal states of polarization (SOPs), one vertical and one horizontal. The two SOPs are then coded and user data are uploaded to the two coded and polarized optical carriers, improving spectrum utilization and increasing the user volume in the system. Moreover, phase-induced intensity noise (PIIN) can be effectively suppressed by combining SAC-OCDMA with polarization division multiplexing (PDM) technology, thereby increasing overall system efficiency [5,6].

However, in the multi-wavelength OCDMA (MW-OCDMA) system, the decoder is sensitive to the relative temporal positioning of optical pulses transmitted simultaneously along different wavelength channels. When the optical fiber transmitting distance of the network is more than 500 m, the system performance will be significantly degraded by chromatic dispersion, which results in temporal skewing among wavelength channels [7]. To improve the distortion, the dispersion compensation scheme is needed in the MW-OCDMA system for compensating dispersion distortion. There are some researches regarding dispersion compensation, such as the following: the compensators that are used to construct a dispersion slope equalizer have been demonstrated in the form of sampled chirped fiber Bragg gratings [8], virtually imaged phased arrays [9], all-pass filters [10], waveguide grating routers with thermal lenses [11], and dispersion compensating fibers (DCFs) [12]. The length of DCFs required to compensate for the dispersion from single-mode fiber (SMF) is long and optical amplifiers will be needed because the insertion loss of DCF is high. Integrated dispersion slope equalizer of AWG-based optical CDMA is then proposed [13]. However, series connections of Mach–Zehnder Interferometers (MZIs) have serious group delay ripples and dispersion compensation is not easier to adjust precisely. Discussion of other issues, such as negative group delay (NGD) at microwave wavelengths, are also presented; the NGD is useful for the compensation of radio frequency/microwave signal delays [14,15].

Orthogonal frequency-division multiplexing (OFDM) is the fastest developing technology in the academic and industrial fields of wireless transmission. OFDM technologies are resistant to frequency-selective interference and demonstrate high transmission rate and high bandwidth usage efficiency. Thus, OFDM is widely applied in broadband communication systems, including three types of fixed-line networks, namely, high-speed digital subscriber line (HDSL), very high-speed digital subscriber line (VDSL), and asymmetric digital subscriber line (ADSL) [16]. Other common applications include digital audio broadcasting (DAB) and broadband wireless access (BWA) systems, as well as the IEEE 802.11 specifications of wireless local area network (WLAN) [17,18,19,20].

In this study, we apply several dispersion equalizer algorithms to construct an electronic dispersion compensation (EDC) equalizer of electrical domain plus with DCF of optical domain which suits for the OFDM-OCDMA with Walsh–Hadamard codes along 10 km-long SMF and 4 km-long DCF. The remainder of this paper is organized as follows. In Section 2, the configuration of the proposed OFDM-OCDMA RoF is described. In Section 3, the effect that chromatic dispersion makes to the OFDM-OCDMA RoF system is introduced. In Section 4, simulation result analysis and compensation mechanism of dispersion compensation fiber DCF and electronic dispersion compensation equalizer in the proposed system is interpreted. Brief conclusions are presented in Section 5.

## 2. System Description of OFDM-OCDMA RoF System

**Figure 1:**
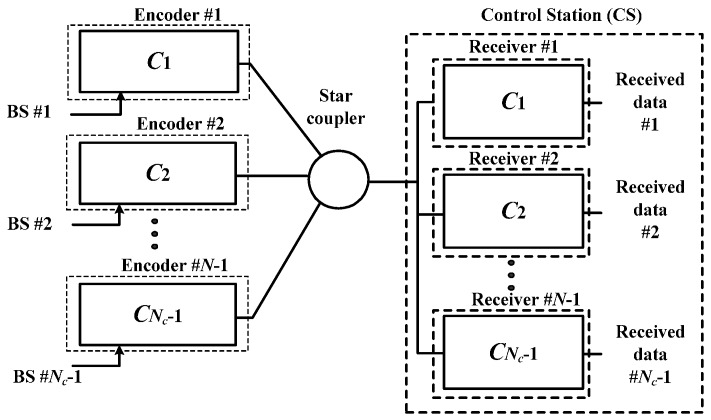
Integrated configuration of orthogonal frequency-division multiplexing-optical code-division multiple-access (OFDM-OCDMA) radio-over-fiber (RoF) system.

Figure 1 presents a block diagram of the proposed OFDM-OCDMA network. In a system architecture, the transmitter largely consists of *N_c_* − 1 base stations (BSs). 

**Figure 2:**
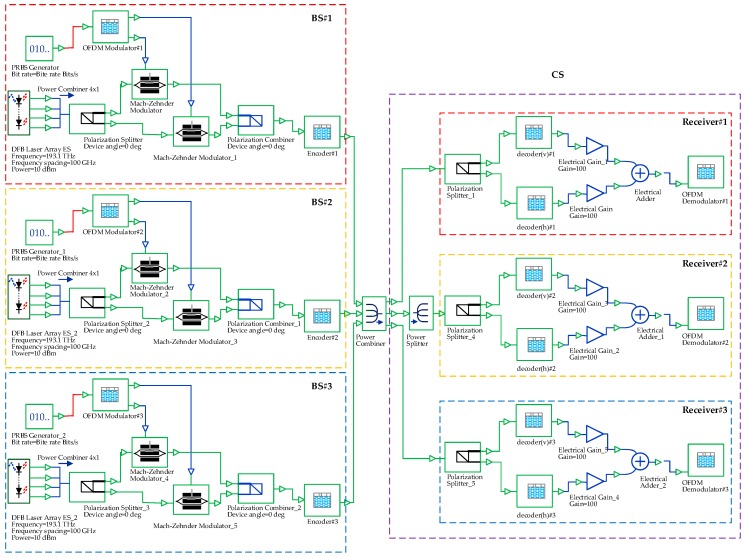
An architectural diagram of an OFDM-OCDMA system with three BSs.

The optical communication simulation software Optisystem 10 which was made by Optiwave Systems Inc., Ottawa, ON, Canada and was used to simulate the proposed system architecture. Figure 2 illustrates a simulation of the OFDM-OCDMA system with three BSs and three receivers in the CS. The BSs loaded the signals of two OFDM channels onto the optical carriers in two SOPs. A polarization combiner was used to combine the polarized signals and transmit the combined signals to an encoder. The encoder coded the signals based on a codeword assigned to each of the BSs. Subsequently, a combiner was used to collect the coded signals, and the signals were transmitted to the CS.

Signals transmitted from the transmitter were the cumulative signals of all active BSs. These signals were transmitted via optical fiber to the receiver. The receiving signals illustrated in Figure 1 of star coupler are the cumulative signals of all the BSs, which can be expressed using the following equation:

(1) $$r(t)=r_{v}(t)+r_{h}(t)=\sum_{k=1}^{K}\left[I_{k}(t)+Q_{k}(t)\right],$$

where *t* is time parameter; $r_{v}(t)$ and $r_{h}(t)$ represent the two receiving signals in the SOPs; $I_{k}(t)$ and $Q_{k}(t)$, respectively, represent the encoded signals of I-channel and Q-channel that were modulated by BSs onto the optical carriers; and *K* represents the number of BSs. However, the present study used the Hadamard code. Therefore, $K=N_{c}-1$, where $N_{c}$ represents the length of the code.

The CS consisted of an equal number of receivers as the number of BSs. Thus, each receiver decoded the signals based on the codeword of one BS. Finally, an OFDM demodulator was used to restore the electrical signals to digital signals.

The output signals in the vertical and horizontal SOPs for Receiver #*l* can be expressed using Equations (2) and (3). The results of the two equations indicate that the proposed method can completely eliminate interference between BSs in ideal conditions.

(2) $$r_{v}(t)\cdot C_{l}-r_{v}(t)\cdot {\bar{C}}_{l}=\{\begin{array}{cc} \frac{N_{c}}{2}\cdot I_{k}(t), & k=l\\ 0, & k\neq l \end{array},$$

(3) $$r_{h}(t)\cdot C_{l}-r_{h}(t)\cdot {\bar{C}}_{l}=\{\begin{array}{cc} \frac{N_{c}}{2}\cdot Q_{k}(t), & k=l\\ 0, & k\neq l \end{array},$$

Finally, the balanced electrical signals in the two SOPs were combined to form complete OFDM signals, which were sent to the OFDM demodulator for demodulation, restoring the electrical signals to digital signals, as expressed in Equation (4).

(4) $$\left[r_{v}(t)\cdot C_{l}-r_{v}(t)\cdot \bar{C}\right]+\left[r_{h}(t)\cdot C_{l}-r_{h}(t)\cdot {\bar{C}}_{l}\right]=\frac{N_{c}}{2}\left[I_{k}(t)+Q_{k}(t)\right],$$

In the system simulations, the present study assumed that the system adopted a back-to-back method to transmit signals. Only the attenuation of the fiber channel was investigated in this section. The effects of CD are discussed in Section 3 and Section 4. Moreover, the OFDM signals transmitted by the wireless transmitter were set at ideal conditions because only the non-ideal effects of the fiber channel were examined in this study. The simulation parameters are tabulated in Table 1.

**Figure 3:**
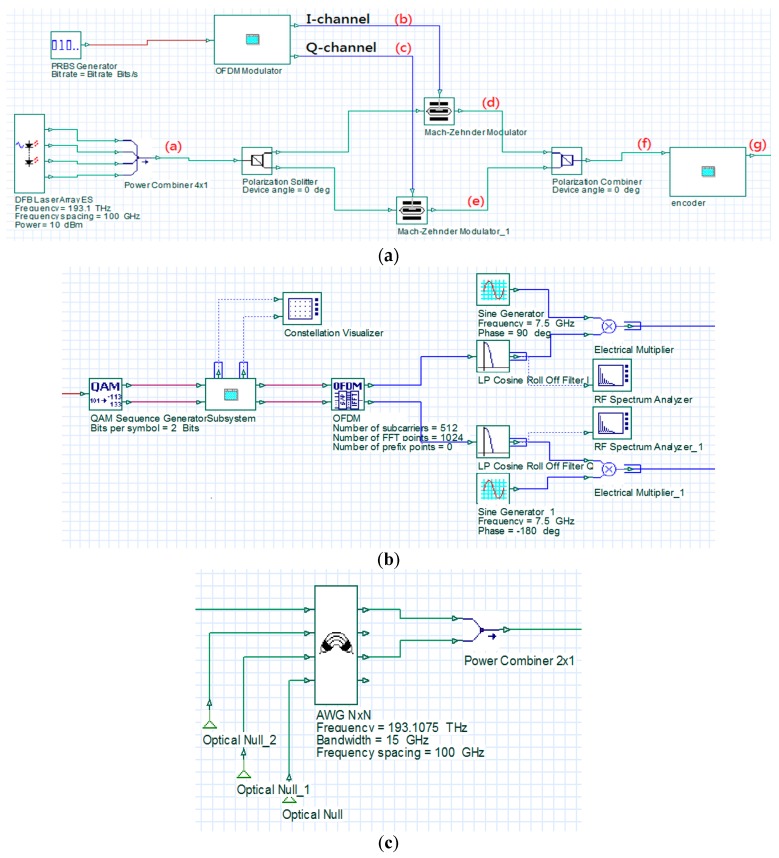
An architectural diagram of the base station (BS) in a simulation environment: (**a**) the base station; (**b**) the OFDM modulator; and (**c**) the OCDMA encoder.

**Figure 4:**
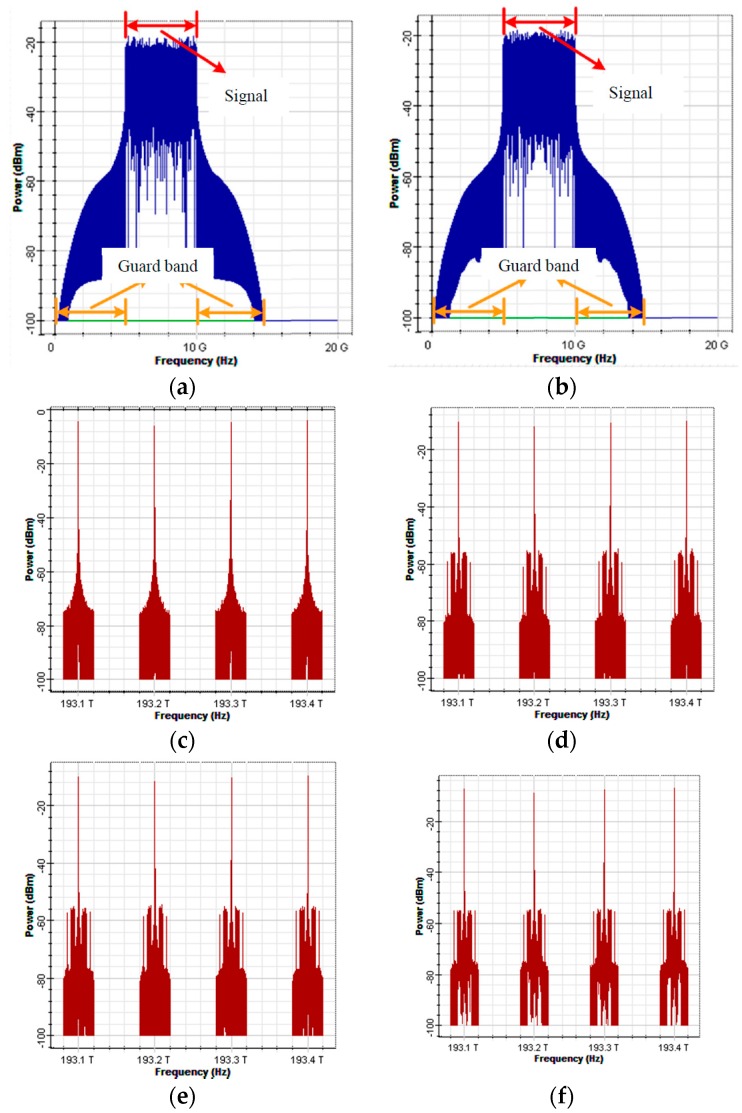

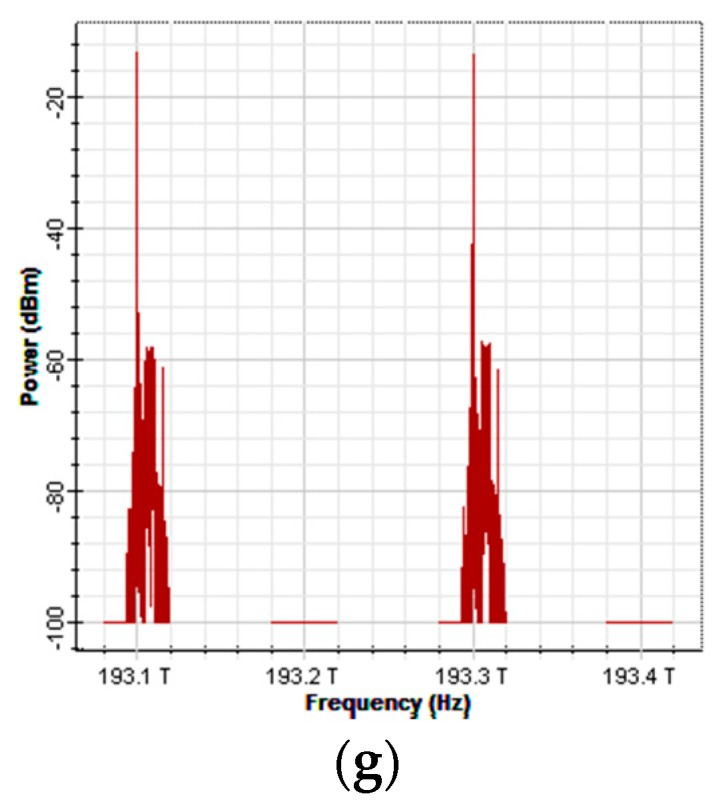
A spectral diagram of the signals at the various nodes of the BS: (**a**) the upscaled signals in I-channel; (**b**) the upscaled signals in Q-channel; (**c**) the optical carrier; (**d**) the modulated, vertically polarized signals; (**e**) the modulated, horizontally polarized signal; (**f**) the combined signals; and (**g**) the encoded signals.

Figure 3 is an architectural diagram of the OFDM modulator and optical encoder in a simulation environment. In Figure 3a; a PRBS generator was used to produce random digital signals with a length of 32,768 bits and a transmission rate of 10 G (bits/s). The signals were then transmitted to the OFDM modulator to produce OFDM signals. In the OFDM modulator, the input digital signals were processed using four-quadrature amplitude modulation (4-QAM). The modulated signals were transmitted to the OFDM block to produce OFDM signals with an inverse fast Fourier transform (IFFT) size of 1024 bits. In order to maintain the wave pattern of the transformed signals consistent with that of the original signals, only the middle 512 carriers were used to load signals. The remaining carriers were loaded with zero signals. The carriers with zero signals imposed no influence on the transformed signals. Then, a low-pass filter was used to de-noise the signals of I-channel and Q-channel produced by the OFDM block. The two baseband signals were separately multiplied by $\cos(2\pi f_{u}t)$ and $-\sin(2\pi f_{u}t)$ to upscale them to 7.5 G. The spectrums of the two baseband signals are illustrated in Figure 4a,b. The overall bandwidth of the signals is 15 G. However, the primary signal range was between 5 and 10 G, and the preceding and succeeding sections (5 G) were the guard intervals.

A distributed feedback laser (DFB) laser array was used to produce four light sources with a linewidth of 5 MHz and central frequencies of 193.1, 193.2, 193.3, and 193.4 THz to serve as the optical carriers (Figure 4c). The optical carriers were polarized into two different SOPs (vertical and horizontal) using a polarization splitter. In addition, an ideal Mach-Zehnder modulator (MZM) was employed to modulate the upscaled signals of I-channel and Q-channel onto the two types of polarized optical carriers. All optical carriers and I-channel and Q-channel signals possess a 5 G guard interval, as illustrated in Figure 4d,e. Figure 4f illustrates a spectral diagram of the combined vertically and horizontally polarized signals using the polarization combiner. Finally, the combiner was used to collect the signals from Output Terminals #1 and #3 of the AWG based on codeword (1010) and the signals were transmitted to the receivers. Figure 4g illustrates a spectral diagram of the encoded signals.

**Figure 5:**
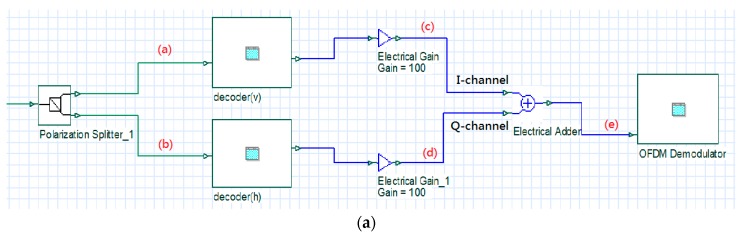

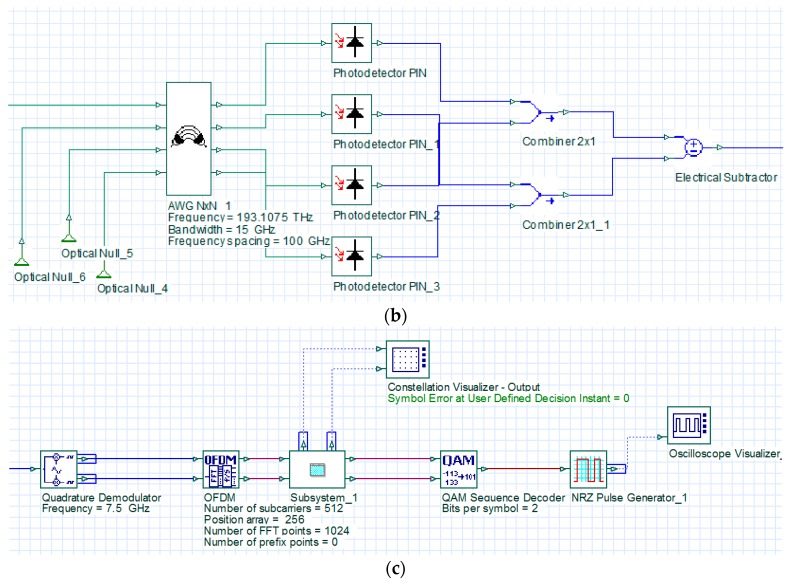
An architectural diagram of a receiver in a simulation environment: (**a**) the architectural diagram of the receiver; (**b**) the OCDMA decoder for the vertical SOP (decoder(v)); and (**c**) the OFDM demodulator.

**Figure 6:**
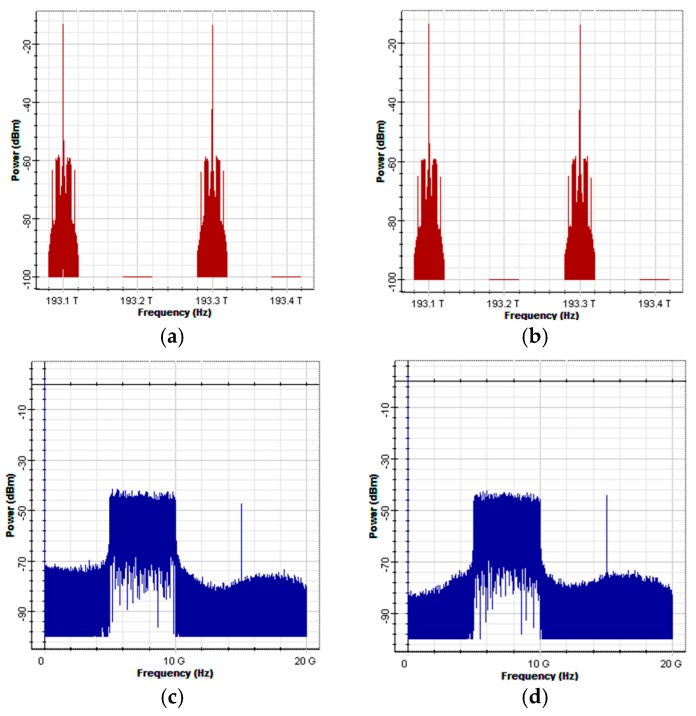

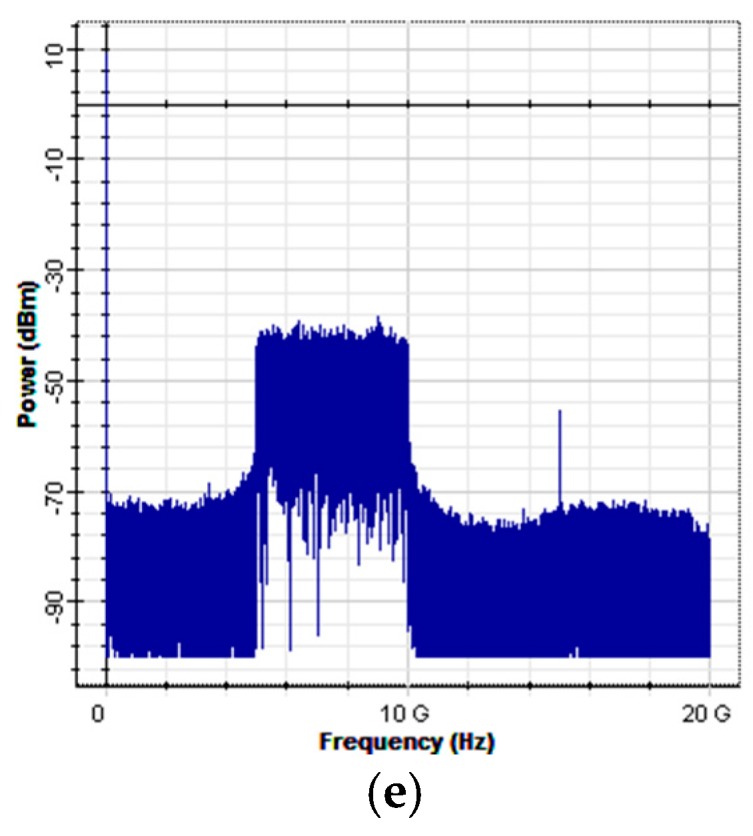
Spectrograms of each node signal: (**a**) vertical SOP signal at the receiver; (**b**) horizontal SOP signal at the receiver; (**c**) I-channel signal after balance detection and amplification; (**d**) Q-channel signal after balance detection and amplification; and (**e**) the complete OFDM signal at the receiver.

Figure 5 illustrates an architectural diagram of a receiver in a simulation environment. First, a polarization splitter was used to split the received signals into vertically and horizontally polarized signals. Then, the two polarized signals were transmitted to the decoder for decoding. Figure 6a,b illustrate the spectral diagrams of the two polarized signals.

In the decoder (using the decoder for the vertical SOP as an example), an AWG was used to filter the optical carrier and the attached OSSB signals. The signals were transmitted to the photodetectors to convert optical data into electrical data. Then, the signals were balanced using the BS codeword. Combiner #1 collected the digital signals from Photodetector #1 and Photodetector #3 ($C_{1}$), and Combiner #2 collected the digital signals from Photodetector #2 and Photodetector #4 (${\bar{C}}_{1}$). The signals of the two combiners were subtracted from one another to eliminate MAI, as illustrated in Figure 5b. The operations of the decoder for the horizontal SOP were similar to those of the decoder for the vertical SOP. Figure 6c,d illustrate the spectral diagrams of the signals in I-channel and Q-channel magnified at 100×, respectively. The signals were magnified 100× before they were transmitted to the OFDM demodulator because after the signals in I-channel and Q-channel were modulated onto the optical carriers and encoded, signal power decreased due to insertion loss, which drastically reduced the power of the signals once they were detected by the receivers. Therefore, the detected electrical signals were magnified 100× to fully display the signal spectrum in the oscilloscope box and to compensate for the loss of signal power during insertion loss and transmission process.

In the OFDM demodulator block, a down-converter was used to downscale the balanced signals to baseband OFDM signals. The downscaled signals were transmitted to a built-in OFDM demodulation block for demodulation, and then to a 4-QAM demodulation block to restore the signals to digital signals, as illustrated in Figure 5c. Finally, the two signals were combined to form complete OFDM signals, which were transmitted to the OFDM demodulator for demodulation. Figure 6e illustrates a spectral diagram of the complete OFDM signals.

In order to address problems of transmission distance, optic fiber was used as the transmission medium. However, this section only investigates the optic fiber simulation results after adding attenuation factors; the simulation results after adding chromatic dispersion (CD) parameters are analyzed in detail in next sections, and a compensation mechanism is also proposed.

To determine accuracy, the present study employed Matlab to process the digital signals of the transmitter modulated through 4-QAM demodulation in an access simulation environment. However, the usable data length was 32,768 due to limited memory, suggesting that when using a single BS with predetermined parameters, the receivers of the OFDM-OCDMA system obtained the minimum bit error rate (BER) measured value is limited in 3 × 10^−5^.

**Figure 7:**
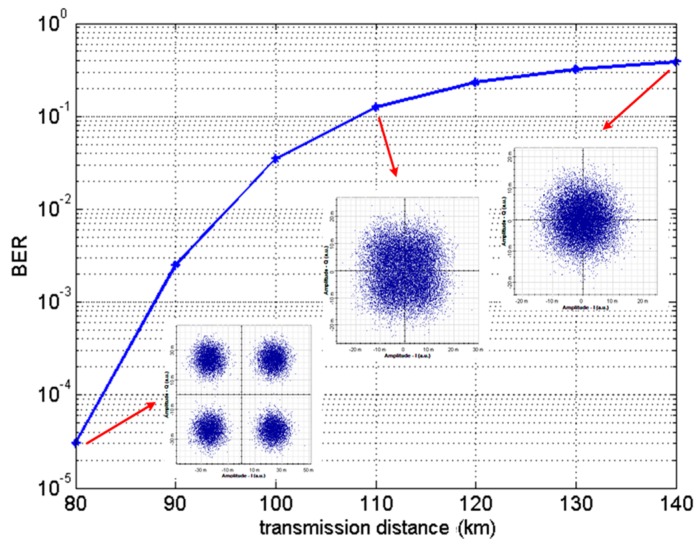
Curve diagram of bit-error-rate (BER) in relation to different transmission distances.

Figure 7 is BER curve diagrams at different transmission distances. Regarding the transmission distance, keeping the input laser source power fixed at 10 dBm and setting the attenuation factor in the optic fiber at 0.2 dB, the BER increases with increased distance. The BER for system architecture with a data length of 32,768 was “0” when the transmitted distance is smaller than 80 km. The cluster points in the constellation diagram also become gradually more blurred. In addition, calculations using the Matlab software indicated that the receiver could completely restore the received signals without errors when the transmission distance was shorter than 80 km.

## 3. Influence of Dispersion on an OFDM-OCDMA System

Dispersion is a type of physical effect caused by different group velocities that generate different time delays when optical carrier waves with different wavelengths are transmitted through an optic fiber. Three main types of dispersion exist, namely modal dispersion, CD, and polarization dispersion. CD can further be divided into material dispersion and waveguide dispersion. In multimode fibers (MMFs), modal dispersion is greater than are material dispersion and waveguide dispersion, making modal dispersion the principal type of dispersion in MMFs. Regarding SMFs, modal dispersion does not exist because only one mode is being transmitted in the optic fiber; only CD and polarization dispersion exist. This study adopts SMF as the transmission medium. Furthermore, the influence of CD in a multi-wavelength system is severer than that of polarization dispersion; therefore, simulation analysis is performed in this section after introducing the dispersion parameters.

The CD is caused by different time delays produced when light with different wavelengths are transmitted in an optic fiber at different velocities. CD can also be divided into material dispersion and waveguide dispersion, and is generally referred to as dispersion. Because actual light sources are not made up of pure monochromatic light and the SMF refraction index varies among light of different wavelengths, the time delay is dissimilar for light of different wavelengths, causing a broadening of the output light pulse. The output pulse expansion is also greater when the optic fiber is longer, which not only causes interference among the data and increases the BER, but also limits the system’s transmission rate and bandwidth. The material dispersion-induced broadening per unit length can be expressed as in Equation (5).

(5) $$\frac{\Delta \tau_{m}}{L}=\left|D_{m}(\lambda )\right|\Delta \lambda ,$$

where the suffix *m* specifies material dispersion, L is the optic fiber length, $\Delta \tau_{m}$ is the pulse expansion time caused by material dispersion, as all light emission sources emit nonmonochromatic light within a specific wavelength range, and $D_{m}(\lambda )$ is the material dispersion coefficient.

Waveguide dispersion is related to the fundamental mode group velocity and the parameter *V*, and *V* is also related to the light source and wavelength. Because light sources have specific frequency spectra, the value of *V* is different when light of different wavelengths is transmitted in the optic fiber, causing the group velocity to differ as well. This further causes the time delay with which each wavelength reaches the end of the optic fiber to differ, inducing a broadening phenomenon of the output signal. Waveguide dispersion differs from material dispersion, in which waveguide dispersion is related to the group velocity and *V*, but the group velocity of material dispersion is related to the refraction index. The pulse broadening per unit length caused by waveguide dispersion is expressed in Equation (6), which is similar to Equation (5).

(6) $$\frac{\Delta \tau_{w}}{L}=\left|D_{w}(\lambda )\right|\Delta \lambda ,$$

where the suffix *w* specifies material dispersion, $\Delta \tau_{w}$ is the pulse expansion time caused by material dispersion and $D_{w}(\lambda )$ is the waveguide dispersion coefficient, which is related to the characteristics of the optic fiber.

The general literature collectively refers to the effects caused by material and waveguide dispersion as dispersion; thus, the total dispersion per unit length can be written as Equation (7).

(7) $$\frac{\Delta \tau_{CD}}{L}=\left|D_{m}(\lambda )+D_{w}(\lambda )\right|\Delta \lambda ,$$

where $\Delta \tau_{CD}$ is the output pulse expansion time caused by dispersion. 

As previously described, different wavelengths of light reach the receiver at distinct times. Therefore, when the OFDM signal has been carried onto the optical carrier wave, each subcarrier signal of OFDM respectively represents an optical carrier wave, and will cause the signals carried on each subcarrier to reach the receiver at different times after transmission through the optic fiber.

**Figure 8:**
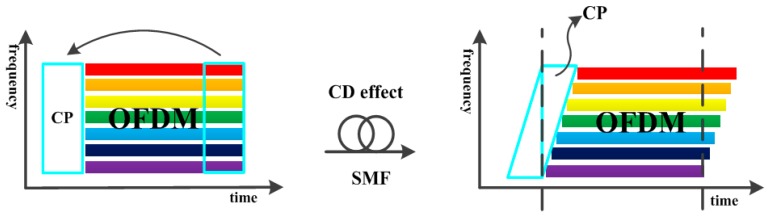
Schematic diagram of dispersion-induced ISI effect.

Figure 8 shows that if no cyclic prefix is added, the receiver will lack part of the signal when it retrieves the signal. Furthermore, if the receiver retrieves other OFDM signals, an intersymbol interference (ISI) effect is generated, greatly reducing the system’s effectiveness. The different colors mean different frequencies of modulated OFDM signal. By adding a cyclic prefix in the OFDM technology, the OFDM signal data loss at the receiver can be compensated and the influence of ISI on the signal can be suppressed. In a related study, scholars utilized a single optical carrier wave to transmit OFDM data, and they deduced the cyclic prefix time length required by OFDM signals to suppress dispersion-induced ISI [21]. However, because the present system principally utilizes multiple optical carrier waves to transmit data, the method of adding a cyclic prefix alone cannot be used to suppress ISI and dispersion-induced interference.

In general, the use of DCF to compensate for dispersion was adopted in the proposed system. In addition, because most of the dispersion is suppressed by the DCF, the length of the cyclic prefix added to the OFDM signal in this system architecture only needs to be identical to the length used in general OFDM technology (e.g., 1/4 of the inverse fast Fourier transform (IFFT) size).

## 4. Simulation Result Analysis and Compensation Mechanism

In this section, analysis is conducted on the simulation results of the OFDM-OCDMA system after adding the dispersion parameters, and the compensation ability of the proposed EDC equalizer is investigated. The SMF simulation parameters are presented in Table 2.

The parameters in Table 2 were selected mainly by referring to the SMF specifications set by the International Telecommunication Union and the specification to the SMF-28-J9 sold by Thorlabs [22,23].

**Figure 9:**
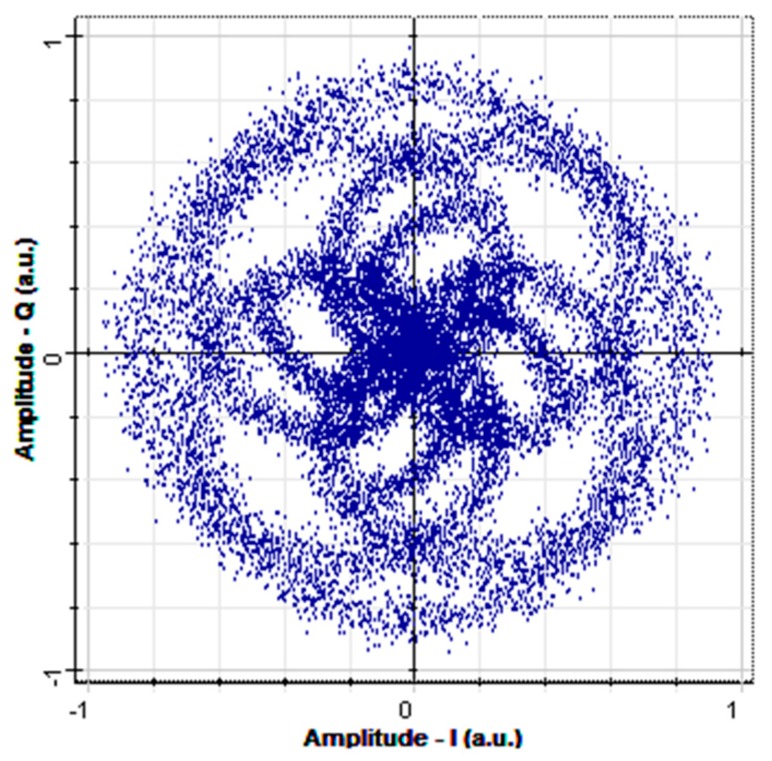
Receiver constellation diagram after 10 km of SMF transmission.

Figure 9 is a receiver constellation diagram of an optical signal after having been subjected to 10 km SMF transmission and dispersion influence. The unit a.u. in Figure 9 means arbitrary units. After calculation, the BER of this constellation diagram was found to be 0.485. The transmitted optical signal causes the receiver constellation diagram to become blurred, making it impossible to modulate the increased error rate (Figure 9).

The main reason causing the OFDM-OCDMA system receiver constellation diagram to become blurred and concentrated is that this system utilizes multiple optical carrier waves to transmit the OFDM signal, and optical carrier waves of different wavelengths that are transmitted through an optic fiber will reach the receiver at different times because of dispersion. In addition, in the OFDM-OCDMA system, the OFDM signals entrained in each optical carrier wave are also influenced by dispersion, causing the signals to reach the receiver at inconsistent times. Therefore, when the receiver performs signal collection and balance detection on the two polarization states, the phase signals of the I-channel and Q-channel will be destroyed because of frequency dispersion. In this type of situation, simply using EDC equalizer technology cannot compensate for the signal. This demonstrates that compensation cannot be performed directly on the electrical signal when transmitting optical signals in an OFDM-OCDMA system. Therefore, compensation must first be performed in the initial stage of the optical transmission domain; the dispersion that could not be fully compensated is subsequently compensated using EDC equalizer technology.

Regarding the influence of dispersion, this study adopted DCF to compensate for dispersion in the optical domain. The simulation parameters are presented in Table 3.

The parameters in Table 3 were selected primarily by referring to the specification to the DCF with model number DCF38 sold by Thorlabs [24]. In addition, the system’s total dispersion amount can be obtained using Equation (8).

(8) $$D_{all}=D_{SMF}\cdot L_{SMF}+D_{DCF}\cdot L_{DCF},$$

where $D_{all}$ is the system’s total dispersion amount; $D_{SMF}$ and $D_{DCF}$ are the SMF and DCF dispersion values, respectively; $L_{SMF}$is the SMF transmission distance; and $L_{DCF}$ is the required DCF length.

**Figure 10:**
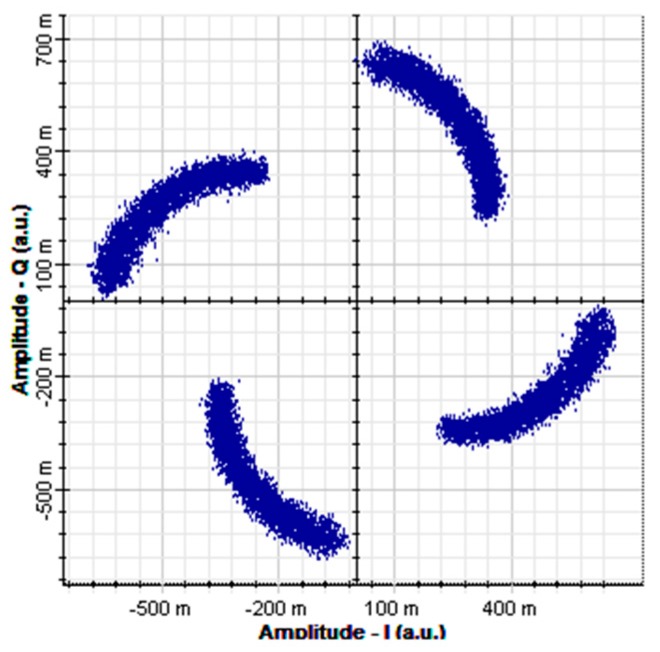
Receiver constellation diagrams after DCF compensation (DCF length is set to 4 km).

In addition, assuming a situation where DCF cannot completely compensate for the dispersion, setting the SMF and DCF lengths to respectively 10 km and 4 km and subsequently utilizing Equation (8) for calculation, the system’s total dispersion amount can be found to be 16 ps/nm. The constellation diagram obtained after simulation is shown in Figure 10, and the BER is 0.5 in this situation.

Regarding situations where the dispersion has not been completely compensated, the insertion of pilot symbols was used to conduct optic fiber channel estimation and compensate for the phase shift. First, the received signal was assumed to be as expressed in Equation (9).

(9) $$r_{ik}=x_{ik}⊗h_{k}+n_{ik},$$

where *i* represents the *i*th data symbol, *k* is the *k*th subcarrier, $r_{ik}$ is the received signal, $x_{ik}$ is the transmitted signal, $h_{k}$ is the fiber channel effect, and $n_{ik}$ is random noise. Subsequently, a least-square algorithm was used to estimate the frequency response in the fiber channel, as shown in Equation (10), where ${\hat{H}}_{ik}$ equals FFT$\left\{h_{k}\right\}$.

(10) $${\hat{H}}_{ik}=X_{ik}^{-1}\cdot R_{ik}=X_{ik}^{-1}\cdot (X_{ik}\cdot H_{ik}+N_{ik})=H_{ik}+X_{ik}^{-1}\cdot N_{ik},$$

**Figure 11:**
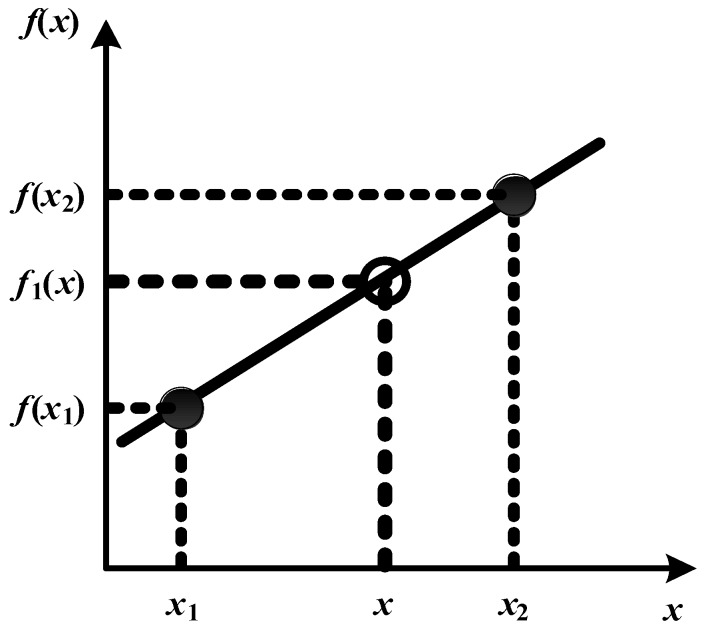
Schematic diagram of the linear interpolation method.

Regarding the pilot symbol arrangement, a comb-type arrangement was adopted; pilot symbols were periodically inserted on the subcarriers and then removed at the receiver after they had been subjected to channel interference, and the calculation was performed. For the subcarriers without pilot symbols, the interpolation method was used to estimate the channel’s frequency response. This study primarily adopted a linear interpolation method to conduct channel estimation. The schematic diagram is shown in Figure 11 and the interpolation value can be obtained using Equation (11).

(11) $$f_{1}(x)=f(x_{1})+\frac{f\left(x_{2}\right)-f\left(x_{1}\right)}{x_{2}-x_{1}}\left(x-x_{1}\right),$$

In Equation (11), the point slope form is utilized to calculate the estimated numerical value between two points. Furthermore, the estimation results are typically more accurate the smaller the interval between two data points is in situations when the linear interpolation method is used.

**Figure 12:**
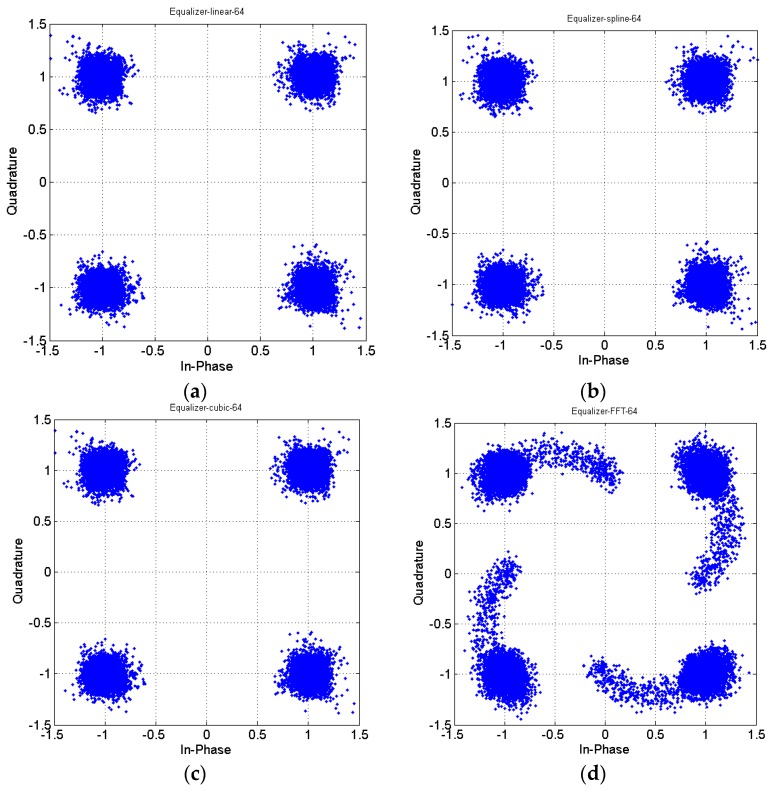
Constellation diagrams after estimation and equalization of fiber channels using different interpolation methods: (**a**) linear interpolation; (**b**) piecewise cubic spline interpolation; (**c**) piecewise cubic Hermite interpolation; and (**d**) fast Fourier transform (FFT) interpolation.

Figure 12 shows a receiver constellation diagram after 10 km of SMF transmission and 4 km of DCF transmission, with no optical amplifier added and after electrical equalization. One pilot symbol is inserted at intervals of 64 subcarriers. In addition, a cyclic prefix with a length of 1/32 of the IFFT amount is added to suppress ISI. A comparison of Figure 10 and Figure 12 indicates that after compensation through linear interpolation equalization, the cluster points in the constellation diagram became relatively converged and exhibited no phase shift. In addition to adopting linear interpolation technology, this study also investigated three common equalizer technologies, namely piecewise cubic spline interpolation, piecewise cubic Hermite interpolation, and FFT interpolation. On the other hand, the constellation diagrams using the linear, piecewise cubic spline, and piecewise cubic Hermite interpolation methods for channel estimation are highly similar after equalization, and they possess identical effectiveness, as shown by Figure 12a–c.

Conversely, the constellation diagram obtained by using the FFT interpolation method is relatively diverged, as indicated by Figure 12d. The main reason for these results is that signals are subjected to influence from different factors in different transmission channels. For example, in a wireless channel, the signal is affected by environmental factors and interference from barriers, while a signal transmitted in an optic fiber is influenced by interference from dispersion and other factors. Perhaps using the FFT interpolation method can achieve excellent effectiveness in wireless channels, but the same effectiveness might not be attainable in fiber channels; therefore, the appropriate interpolation method should be selected according to various channel effects.

In addition, because of the assumption that the DCF could not completely suppress the dispersion, the received signal will be influenced by small amounts of dispersion. That is why the pilot symbol insertion spacing was increased to 64, and a cyclic prefix with the length 32 (generally 1/4 of the IFFT size) was used to compensate for the dispersion-induced ISI. Regarding the interpolation methods, because the system effectiveness obtained by using linear, piecewise cubic spline, and piecewise cubic Hermite interpolation was identical, the linear interpolation method was adopted as an equalizer technology in the OFDM-OCDMA compensated scheme. The main reason for selecting the linear interpolation method for channel estimation in this study was because the associated calculation amount is relatively small.

In Figure 12, we can find that proposed dispersion equalizer scheme of electrical domain plus with DCF of optical domain is effectively suppressing CD effect and hence improving system performance.

## 5. Conclusions

This study investigated the dispersion effects in an OCDMA system combined with a microwave optic fiber network using OFDM and polarization multiplexing technology. In the system architecture presentation, the encoding and decoding architecture utilized arrayed waveguide grating to code the frequency domain amplitude. Subsequently, the design parameters of the arrayed waveguide grating were changed to change the bandwidth and frequency and thus enable the system to transmit two different signal formats (i.e., unilateral and bilateral optical carriers). In addition, polarization multiplexing technology was added into the BS and receiver architecture to suppress interference caused by phase-induced intensity noise and thus enhance the system’s overall effectiveness. In addition, when the two signals were separately transmitted through the optic fiber in a situation where only influence from attenuation factors was considered and the BER equaled 3 × 10^−5^, the transmission distance could reach 80 km.

When the long distance transmission dispersion effect was added, the signal could no longer be restored after a 10-km transmission. In this study, an ideal effect was initially investigated, and DCF plus with electrical equalizer was used to compensate for dispersion. After the dispersion effect was fully compensated using the DCF, the simulation results indicated that the signal could be restored effectively and that the BER equaled zero when the transmitted data length was 32,768. When the DCF is unable to completely compensate for the dispersion, the received signal will still be affected by small amounts of dispersion and therefore the cluster points in the constellation diagram will exhibit phase shifting, causing the system’s effectiveness to decline. In this situation, the present system utilized OFDM equalizer technology to insert pilot symbols to perform estimation and compensation on the fiber channel. The simulation results showed that this method can effectively suppress interference of the signal from remaining dispersion when the DCF could not attain complete compensation. The method also enables effective restoration of the transmitted signal and enhancement of overall system effectiveness.

## Figures and Tables

**Table 1:** The Simulation Parameters.

Modulation Technique	4-QAM
Data Length	32,768 bits
Data Transmission Rate	10 G bits/s
IFFT Size	1024
Upscale Frequency (*f_u_*)	7.5 GHz
Electronic Filter Bandwidth (*B*)	6 GHz
Input power	10 dBm
Number of Optical Carriers	4
Laser Beam Width	5 MHz
Optical Carriers Spacing	100 GHz
AWG Bandwidth	15 GHz

**Table 2:** SMF simulation parameters.

Wavelength	1550 nm
Attenuation	0.2 dB/km
Dispersion (*D_SMF_*)	17 ps/nm × km
Dispersion slope	0.056 ps/nm^2^ × km

**Table 3:** DCF simulation parameters.

Wavelength	1550 nm
Attenuation	0.25 dB/km
Dispersion (*D_DCF_*)	−38.5 ps/nm × km
Dispersion slope	−0.12 ps/nm^2^ × km

## References

[1] Wu J.S., Wu J., Tsao H.W. (1998). A radio-over-fiber network for microcellular system application. IEEE Trans. Veh. Technol..

[2] Al-Raweshidy H., Komaki S. (2002). Radio over Fiber Technologies for Mobile Communication Networks.

[3] Kavehrad M., Zaccarin D. (1995). Optical Code-Division-Multiplexed Systems Based on Spectral Encoding of Noncoherent Sources. IEEE J. Lightwave Technol..

[4] Huang J.F., Hsu D.Z. (2000). Fiber-grating-based optical CDMA spectral coding with nearly orthogonal M-sequence codes. IEEE Photonics Technol. Lett..

[5] Yen C.T., Huang J.F., Chang Y.T., Chen B.H. (2010). Polarization diversity scheme on spectral polarization coding optical code-division multiple-access network. Opt. Eng..

[6] Huang J.F., Yen C.T. (2006). Phase noise suppression in multilevel optical code-division multiple-access network coding system with embedded orthogonal polarizations. Opt. Eng..

[7] Ng E.K.H., Weichenberg G.E., Sargent E.H. (2002). Dispersion in Multiwavelength Optical CDMA Systems: Impact and Remedies. IEEE Trans. Commun..

[8] Cai J.X., Feng K.M., Willner A.E., Grubsky V., Starodubov D.S., Feinberg J. (1999). Simultaneous tunable dispersion compensation of many WDM channels using a sampled nonlinearly chirped fiber Bragg grating. IEEE Photonics Technol. Lett..

[9] Shirasaki M. (1997). Chromatic dispersion compensator using virtually imaged phased array. IEEE Photonics Technol. Lett..

[10] Madsen C.K., Lenz G., Bruce A.J., Cappuzzo M.A., Gomez L.T., Scotti R.E. (1999). Integrated all-pass filters for tunable dispersion and dispersion slope compensation. IEEE Photonics Technol. Lett..

[11] Doerr C.R., Stulz L.W., Chandrasekhar S., Pafchek R. (2003). Colorless tunable dispersion compensator with 400-ps/nm range integrated with a tunable noise filter. IEEE Photonics Technol. Lett..

[12] Vengsarkar A.M., Reed W.A. (1993). Dispersion-compensating single-mode fibers: Efficient design for first- and second- order compensation. Opt. Lett..

[13] Yen C.T. (2010). Integrated dispersion slope equalizer of AWG-based optical CDMA for radio-over-fiber transmissions. Photonic Netw. Commun..

[14] Ravelo B. (2011). Investigation on microwave negative group delay circuit. Electromagnetics.

[15] Ravelo B. (2014). Distributed NGD active circuit for RF-microwave communication. Int. J. Electron. Commun. (AEÜ).

[16] Bingham J.A.C. (2000). ADSL, VDSL, and Multicarrier Modulation.

[17] ETSI (1995). Radio Broadcasting System, Digital Audio Broadcasting (DVB) to Mobile, Portable and Fixed Receiver.

[18] Hoeg W., Lauterbach T. (2000). Digital Audio Broadcasting: Principle and Applications.

[19] Reid N.P., Seide R. (2002). 802.11(Wi-Fi) Networking Handbook.

[20] IEEE Standards Department (1997). IEEE 802.11 Draft Standard for Wireless LAN Medium Access Control (MAC) and Physical Layer (PHY) Specification.

[21] Shieh W., Athaudage C. (2006). Coherent optical orthogonal frequency division multiplexing. Electron. Lett..

[22] International Telecommunication Union Characteristics of a Single-Mode Optical Fiber and Cable. https://www.itu.int/rec/T-REC-G.652/en.

[23] Thorlabs SMF-28-J9 Spec Sheet. https://www.thorlabs.com/thorproduct.cfm?partnumber=SMF-28-J9.

[24] Thorlabs DCF-38 Spec Sheet. https://www.thorlabs.com/thorproduct.cfm?partnumber=DCF38.

